# Ebstein’s Anomaly in Disguise: Follow the Cues and the Diagnosis Can Be Made

**DOI:** 10.7759/cureus.10773

**Published:** 2020-10-02

**Authors:** Tanya Sharma, Fuad Habash, John Mounsey, Chris Baker, Angel Lopez Candales

**Affiliations:** 1 Internal Medicine, University of Arkansas for Medical Sciences, Little Rock, USA; 2 Cardiology, University of Arkansas for Medical Sciences, Little Rock, USA; 3 Cardiac Noninvasive Laboratory, University of Arkansas for Medical Sciences, Little Rock, USA

**Keywords:** ebstein’s anomaly, tricuspid regurgitation, arvc, epsilon wave

## Abstract

Ebstein’s anomaly is a congenital defect, which is rarely present in adults with arrhythmias and right heart failure with tricuspid regurgitation. The diagnosis is made by non-invasive cardiac imaging with transthoracic echocardiography or cardiac magnetic resonance imaging. However, mild and atypical anatomical variants require a more specific investigation to make the diagnosis and differentiate it from other pathologies which have a similar presentation, including Arrhythmogenic Right Ventricular Cardiomyopathy (ARVC). We present the case of a 66-year-old gentleman with a history of recurrent admissions due to decompensated heart failure exacerbations, now presenting with gradually worsening dyspnea. An echocardiogram was obtained, showing a thin-walled, dilated, and dysfunctional right ventricle (RV) with severe tricuspid regurgitation due to poor coaptation of the tricuspid leaflets. Although a very distinctive epsilon wave was seen on his electrocardiogram, highly suggestive of arrhythmogenic RV cardiomyopathy (ARVC), which would be enough to explain his presentation and initial echocardiogram; an off-axis plane of the tricuspid valve without any RV aneurysm or dilation of the RV outflow tract was incongruent with this diagnosis. Additional echocardiographic images were determinant to demonstrate both apical displacement and tethering of the septal tricuspid leaflet with an abnormally long anterior tricuspid leaflet, suggestive of Ebstein’s anomaly. This diagnosis was confirmed with cardiac magnetic resonance imaging. Mild variants of Ebstein’s anomaly, especially in the presence of confounding findings require focused imaging to ascertain the diagnosis. We review these non-traditional findings in trying to differentiate Ebstein’s from ARVC.

## Introduction

Ebstein’s anomaly is a rare congenital heart disorder affecting 1 in every 200,000 live births, accounting for less than 1% of all congenital heart disease cases [1,2]. First described by Wilhem Ebstein in 1866 [3], this malformation is characterized by (a) tethering of the septal and posterior leaflets to the underlying myocardium, (b) apical displacement of the tricuspid valve (TV) plane more prominent for the septal leaflet and in descending order, the posterior and then the anterior leaflet, (c) dilation of the right ventricle (RV) due to contribution of a superior atrialized portion of the right atria with various degrees of hypertrophy and thinning of the wall, (d) abnormal elongation with fenestrations of the anterior tricuspid leaflet, and (e) dilatation of the tricuspid annular plane [4]. 

In terms of nomenclature and classification, Carpentier et al, first proposed that Ebstein’s anomaly can be divided as: Type A in which the volume of the true RV is adequate; Type B in which a large atrialized portion of the RV is present but the anterior tricuspid leaflet moves freely; Type C in which the anterior leaflet is severely restricted, causing RV outflow tract obstruction; and Type 4 in which only a small infundibular portion of the RV is not involved by massive atrialization [5]. 

Consequently, echocardiographic distinction was then proposed by Celermajer et al based on corresponding abnormalities between the combined size of the right atrium and ventricle and the left ventricle as follows: Grade 1 ratio less than 0.5, Grade 2 ratio between 0.5 to 0.99, Grade 3 ratio between 1.0 and 1.49 and Grade 4 ratio 1.5 or larger [6]. Although the etiology of Ebstein’s anomaly remains largely unresolved, familial recurrence and genetic mutations may account for a small proportion of all cases with other incidences associated with fetal exposure to benzodiazepines and lithium [7,8]. 

While most patients present themselves in infancy and childhood, milder isolated Ebstein’s anomaly may be diagnosed in adulthood. Typical presentation in adolescents and adults include arrhythmias (in 40% cases) and occasionally, right heart failure symptoms [6,9,10]. Other pathologies that present with tricuspid regurgitation include arrhythmogenic right ventricular cardiomyopathy (ARVC), carcinoid syndrome, congenital tricuspid valve atresia, tricuspid valve prolapse, tricuspid valve endocarditis and functional tricuspid regurgitation due to impaired right ventricular geometry secondary to ischemic or other downstream causes like pulmonary hypertension and left heart failure [11].

Diagnosis is made by visualization of the valvular apparatus and right ventricular anatomy with transthoracic echocardiography (TTE). Further evaluation can be done with cardiac magnetic resonance imaging (MRI), which can provide better quantification of RV size and function [12]. Cardiac MRI is also useful in locating fibrosis (late gadolinium enhancement) which can be focal or diffuse in the right atrium (RA) and RV of patients with Ebstein’s anomaly as well as quantifying it (with pre- and post-contrast T1 mapping), which is related with disease severity and prognosis [13,14].

However, mild or incomplete variants of the anomaly pose a diagnostic difficulty that may lead to delay in diagnosis, as in this case it was delayed for 4 years until appropriate echocardiographic images were obtained and led to confirmation with cardiac MRI.

## Case presentation

A 66-year-old gentleman was admitted with progressive shortness of breath and bilateral lower extremity edema. His past medical history was remarkable for congenital hydrocephalus with a ventriculo-peritoneal (VP) shunt, chronic right lower extremity lymphedema from orthopedic surgery, atrial flutter and heart failure with reduced ejection fraction with reportedly poor medication compliance.

Over the previous four years, the patient had multiple hospital admissions for shortness of breath and atypical chest pain. In 2016, he was diagnosed with typical counterclockwise atrial flutter. In 2017, he once again presented with shortness of breath but this time he had atrial fibrillation and underwent electrical cardioversion, with atrial flutter recurrence in 2018 requiring atrial flutter ablation. At that time, worsening left ventricular function was noted with progressive right ventricular dilation and varying degrees of tricuspid regurgitation. Investigation into anatomic and secondary causes of presentation was inconclusive.

**Figure 1:**
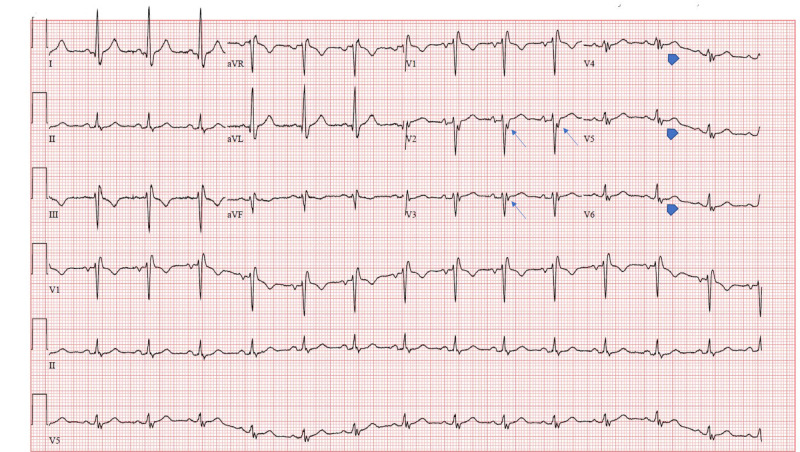
ECG with Epsilon waves in V2 and V3 (arrows). However, a similar signal was also seen in V4-V6 (arrowhead)

During the current admission, the patient was hemodynamically stable but tachycardic (100 bpm) and saturating well at room air. Physical examination was significant for jugular venous distension to mid neck with positive hepatojugular reflux and bilateral crackles with +4 pitting lower extremity edema up to bilateral thighs. BNP (Brain natriuretic peptide) was elevated (665 pg/mL; Ref: </=100 pg/mL), but troponin was negative. ECG showed sinus tachycardia, non-specific intraventricular conduction delay and epsilon waves in leads V2-V6 (Figure 1). 

**Figure 2:**
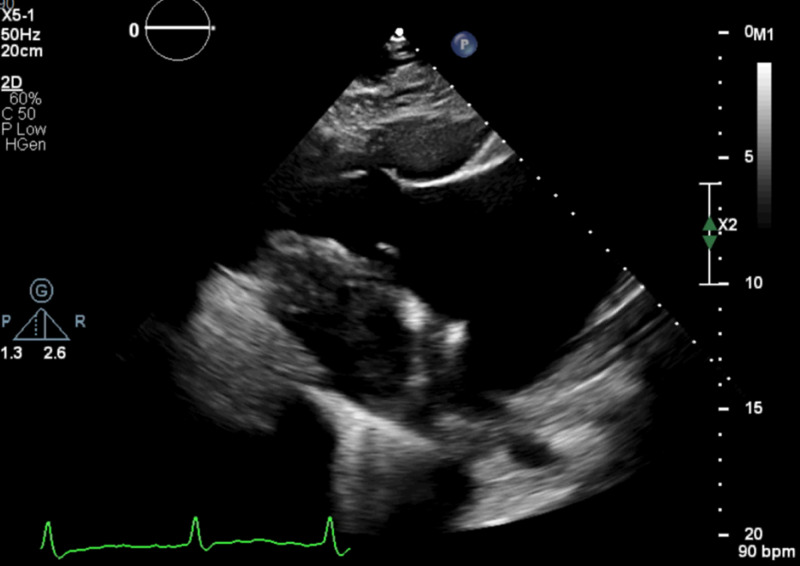
Echocardiographic image with wide open tricuspid valve with minimal visualization of leaflets

**Figure 3:**
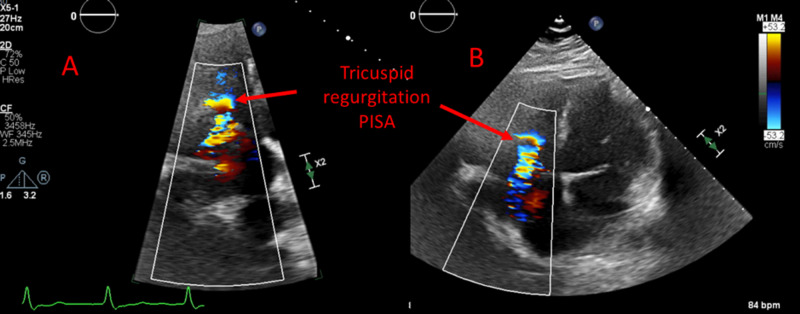
Echocardiography with color doppler: Severe tricuspid valve regurgitation with large Proximal Isovelocity Surface Area (PISA)

An initial limited TTE showed left ventricular ejection fraction (LVEF) of 40% with mild diffuse hypokinesis, marked dilation of the right atrium (RA) and RV with decreased RV systolic function (TAPSE (tricuspid annular plane systolic excursion) 1.1 cm and TDI (tissue doppler imaging) 5 cm/s). In addition, from the inflow view the posterior leaflet showed limited motion while the anterior leaflet was not well seen (Figure 2), leading to significant tricuspid regurgitation (Figure 3) without evidence of pulmonary hypertension. 

**Figure 4:**
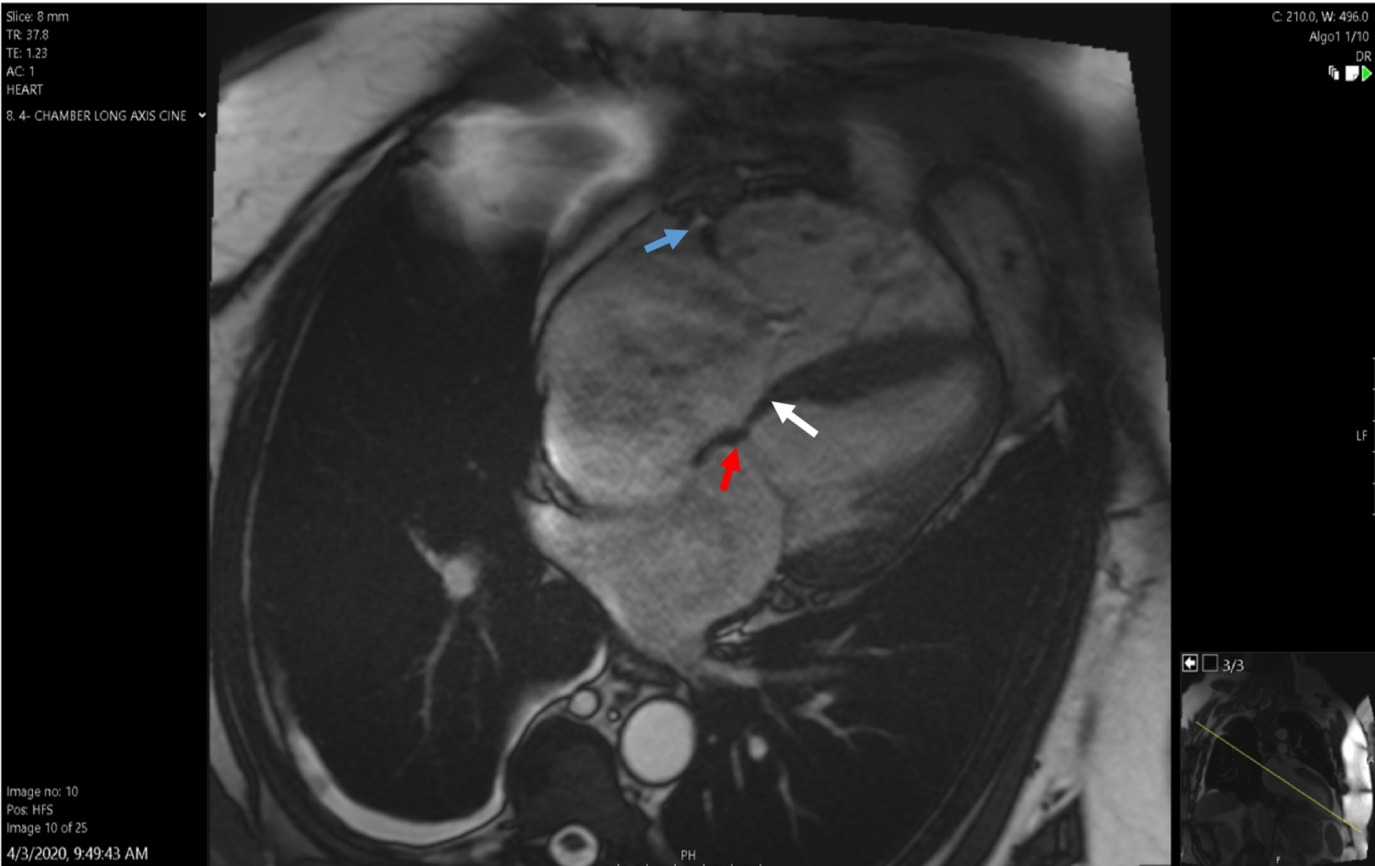
Cardiac MRI in four-chamber view showing the apically displaced septal tricuspid leaflet (white arrow) in relation to the mitral annulus (red arrow). Anterior tricuspid leaflet shows a normal annular insertion (blue arrow) but the coaptation point is lower in the right ventricle

Since not all echocardiographic findings were congruent, particularly with the epsilon wave on ECG, a cardiac MRI was obtained. The MRI showed marked dilation of the RA and moderately dilated RV (RV end diastolic volume 143.6 ml/m2; normal 50-105 ml/m2) with decreased RV ejection fraction (37.5%). The RV/LV volume ratio was 2:1. No RV wall dyskinesia, aneurysm or fat deposition was seen. There was severe tricuspid regurgitation (Figure 4). No late gadolinium enhancement was noted on phase sensitive inversion sequence. There were no other findings that would indicate ARVC on MRI. Since the patient had diarrhea and some facial flushing, we considered carcinoid syndrome as a differential diagnosis but the urinary 5-Hydroxyindoleacetic acid (5-HIAA) level was negative.

The patient improved with intravenous diuresis. A dedicated TTE was then repeated using nonstandard imaging planes and for the first time, the point of coaptation and resultant tricuspid regurgitation was found within the mid RV cavity. In addition, the septal tricuspid leaflet was tethered and apically displaced (26mm from the mitral counterpart). The origin of the anterior leaflet was normally located but the point of coaptation was also apically displaced (20 mm into the main RV chamber) (Figure 4). Severe regurgitation was seen with flow reversal into the hepatic veins. 

Based on these imaging findings, we arrived at a diagnosis of a variant of Ebstein’s anomaly. The patient underwent right heart catheterization for hemodynamic assessment, which was significant for raised right sided pressures (mean right atrial pressure 19 mmHg, right ventricular pressure 34/15 mmHg, pulmonary artery pressure 35/25 mmHg with mean of 28 mmHg), pulmonary capillary wedge pressure (21 mmHg) and decreased cardiac index (1.78 L/min/m2). The patient was discharged after optimization of fluid status with appropriate medical therapy for heart failure and close follow-up in clinic, with plan for surgical evaluation by an adult-congenital cardiac surgeon.

## Discussion

Ebstein’s anomaly is a relatively rare congenital cardiac malformation that can pose serious management challenges from both a hemodynamic and electrophysiological perspective. Its etiology remains uncertain and both genetic and environmental factors have been implicated. The central anatomic derangement is characterized by an antero-caudal displacement of the TV annulus and atrialization of the RV, leading to right heart dysfunction and tricuspid regurgitation of varying degrees. Milder forms may present later in adulthood, however less than 5% of adults survive to the fifth decade of their life without heart failure [6]. The defect is caused due to failure of delamination during embryonic development of the heart, leading to adherence of the septal and posterior leaflets to the underlying myocardium [10].

Diagnosis is made by measuring the apical displacement of septal and posterior TV leaflets in relation to the anterior mitral valve leaflet. A displacement of 8 mm of more per body surface area is diagnostic [10]. The apical displacement index was noted to be 11.8mm/m2 in our patient. However, since the displacement was not apparent in any of the echocardiograms from the last four years, we had to consider a broad differential and pursue more diagnostic tests.

Amongst other likely diagnoses, carcinoid syndrome was ruled out due to inconsistent systemic symptoms and negative 24 hour urinary collection of 5-HIAA [15]. ARVC was another possible diagnosis and it is indeed more prevalent than Ebstein’s anomaly in the general adult population (1 in 5000) [16]. ARVC is characterized by localized or generalized dilatation of the RV along with thinning of the myocardium and has a distinctive finding of an epsilon wave on ECG, hence being on our differential list. The epsilon wave is a distinct low amplitude signal between the end of the QRS complex to the onset of the T‐wave in the right precordial leads V1‐V3 [17]. Progressive biventricular failure, as seen in our patient, is a finding in 56% of patients affected by ARVC [18]. 

Cardiac MRI findings of ARVC include global and regional ventricular dilatation, ventricular dysfunction, intramyocardial fat deposition, late gadolinium enhancement, focal wall thinning, dis-kinetic bulging and ectasia of the RV outflow tract [19]. Our patient did not have any evidence of the characteristic tachyarrhythmias of ARVC and cardiac MRI was not consistent with the diagnosis in the absence of fatty infiltration or aneurysmal dilatations in the RV wall. 

While the patient met the anatomic diagnostic criteria for Ebstein’s anomaly, the appearance of the myocardium in the functional segment of the RV was thin and lacked visible trabeculations, which is in contrast with what has been popularly described. However, two-thirds of hearts with Ebstein’s anomaly show dilated RV which also involves the functional RV apex and outflow tract [10]. 

With regards to the epsilon wave, it is important to note that it represents fragmentation of the QRS complex due to delayed potentials resulting from slow intraventricular conduction from islands of surviving myocardium interspersed with fatty and / or fibrous tissue. It has been identified in numerous pathological clinical entities besides ARVC [20]. However, Ebstein’s anomaly is not one of these conditions. We believe that the severe RA and RV dilation in our case might have been responsible for the presence of the epsilon wave. 

## Conclusions

Ebstein’s anomaly is a rare diagnosis but should be considered as a differential diagnosis even in older adults who present severe tricuspid regurgitation and predominant right heart failure, in the absence of an alternate explanation. The anatomic variations of the defect may lead to a difficulty and delay in diagnosis. The cardiac MRI and specialized echocardiographic views focusing on the tricuspid valve and right ventricle may be necessary to delineate the anatomy and ascertain the diagnosis.
